# Evaluation of genetic variants using chromosomal microarray analysis for fetuses with polyhydramnios

**DOI:** 10.1186/s12920-022-01224-w

**Published:** 2022-03-30

**Authors:** Xiaoqing Wu, Ying Li, Na Lin, Linjuan Su, Xiaorui Xie, Bing Liang, Qingmei Shen, Meiying Cai, Danhua Guo, Hailong Huang, Liangpu Xu

**Affiliations:** 1 Fujian Provincial Key Laboratory for Prenatal Diagnosis and Birth Defect, Medical Genetic Diagnosis and Therapy Center of Fujian Provincial Maternity and Child Health Hospital, Affiliated Hospital of Fujian Medical University, No. 18 Daoshan Road, Fuzhou City, 350001 Fujian Province China; 2 Fujian Provincial Key Laboratory for Prenatal Diagnosis and Birth Defect, Fuzhou City, Fujian Province China; 3 Department of Laboratory Medicine, Fujian Medical University, Fuzhou City, Fujian Province China

## Abstract

**Background:**

Polyhydramnios, the excessive accumulation of amniotic fluid, is associated with an elevated risk of abnormal karyotype, particularly aneuploidy. Studies focusing on chromosomal microarray analysis (CMA) in pregnancies with polyhydramnios are limited. The aim of this study is to evaluate the implications of pregnancy with polyhydramnios by CMA testing and routine karyotyping.

**Methods:**

Data from 131 singleton and 17 twin pregnancies that underwent prenatal CMA testing due to polyhydramnios between May 2017 and May 2021 were reviewed. Enrolled cases were grouped into isolated polyhydramnios (N = 39) and non-isolated polyhydramnios (N = 111). Non-isolated group was further categorized as subgroup of soft markers (n = 59) and non-soft markers (n = 52).

**Results:**

CMA revealed an additional 10 (6.7%) chromosomal aberrations with clinical significance in 9 fetuses from singleton pregnancies and 1 from a twin pregnancy. Six microdeletion/microduplication syndromes were observed, of which 4 were located on chromosome 17. The incremental yields of clinically significant CMA findings in non-isolated polyhydramnios was 8.1%, and the values in fetuses along with soft markers and non-soft markers were 5.1% and 11.5% (*p* > 0.05), respectively. Only one incidental finding related to neuropathy with liability to pressure palsies was detected from 39 fetuses with isolated polyhydramnios.

**Conclusions:**

Non-isolated polyhydramnios is associated with several microdeletion/microduplication syndromes, regardless of singleton or twin pregnancies. Our results suggest insufficient evidence to recommend CMA in pregnancies with isolated polyhydramnios.

**Supplementary Information:**

The online version contains supplementary material available at 10.1186/s12920-022-01224-w.

## Background

Amniotic fluid is derived from the dialysate of maternal serum that enters the amniotic cavity through the fetal membranes, the exudate of fetal lungs, umbilical cord, Wharton’s jelly and fetal skin, as well as fetal urine. In general, amniotic volume increases gradually with the gestation, increasing to about 1000 ml at 36 weeks of gestation and gradually decreasing thereafter. Appropriate amount of amniotic fluid can protect the fetus and the mother. Polyhydramnios refers to the situation that the amniotic fluid volume is more than 2000 mL during pregnancy, with an incidence of between 1–2% [1, 2]. The maximum depth of amniotic fluid (maximum vertical pocket depth, MVP) and amniotic fluid index (AFI) are used for ultrasonic assessment of amniotic fluid volume. The generally accepted definition of polyhydramnios is AFI above 24 cm or MVP above 8 cm [3, 4].

Polyhydramnios could be isolated or accompanied with other ultrasound anomalies. The etiology for polyhydramnios is complicated, with about 60% of them being idiopathic polyhydramnios of unexplained origin, 30% being caused by fetal disease, and the rest being caused by maternal disease and other factors [2]. Polyhydramnios was considered to be related to increased risk of chromosomal aberration mainly in the form of aneuploidy [5, 6]. Thus invasive prenatal testing is routinely recommended for abnormal karyotypes evaluation in many countries. The most frequently detected abnormalities were trisomy 21 and trisomy 18. However, the association between the risk of chromosomal aberrations and isolated polyhydramnios is controversial regarding routine karyotype testing. In last few years, chromosomal microarray analysis (CMA) has been widely applied in the field of prenatal diagnosis, especially for pregnancies with ultrasound anomalies [7–9]. However, studies focusing on the CMA results in pregnancies with polyhydramnios is limited [10, 11]. Therefore, we conducted a retrospective study based on the results of CMA testing and routine karyotyping in pregnancies with polyhydramnios, in order to evaluate the implication value of CMA in pregnancy with polyhydramnios.

## Material and method

### Patients and samples

The retrospective study reviewed 131 singleton pregnancies and 17 twin pregnancies that underwent prenatal CMA testing due to polyhydramnios, accompanied with or without other ultrasound abnormalities between May 2017 and May 2021 at the Medical Genetic Diagnosis and Therapy center of Fujian Maternal and Child Health Hospital, China. All the enrolled pregnant women did not have pregnancies of polyhydramnios before. Polyhydramnios was defined as amniotic fluid index above 24 cm or maximal vertical pocket above 8 cm. Pregnancies with maternal diabetes, isoimmunization, fetal infection, and twin pregnancies with twin-to-twin transfusion syndrome were not included in the study. Among the 17 twin pregnancies, 2 pregnancies showed polyhydramnios in both the twins, and in other 15 pregnancies, polyhydramnios occurred only in one of the twins. As a results, a total of 150 specimens including 81 cases of amniotic fluid and 69 cases of umbilical cord blood were sampled. According to the ultrasound findings, the enrolled 150 fetuses were classified into groups of isolated (N = 39) and non-isolated polyhydramnios (N = 111). Cases with non-isolated polyhydramnios were subgrouped into polyhydramnios associated with soft markers and structural abnormalities. The soft markers included thickened nuchal translucency, thickened nuchal fold, echogenic intracardiac focus, mild ventriculomegaly, choroid plexus cysts, echogenic bowel, mild hydronephrosis, mild tricuspid regurgitation, short femur length, aberrant right subclavian artery, absent or hypoplastic nasal bone, renal pelvis dilatation and single umbilical artery. The mean maternal age was 31 ± 4.7 years old, and the mean gestational age at the diagnosis of polyhydramnios was 27.4 ± 1.9 weeks. Demographic characters were presented in Table 1. The study was approved by the local Ethics Committee of Fujian Maternity and Child Health Hospital. Written informed consent to participate in the study was obtained from each patient.

**Table 1:** Demographic characters for 150 pregnancies with polyhydramnios

	Total (n = 150)	Isolated polyhydramnios (N = 39)	Non-isolated polyhydramnios (N = 119)
Maternal age (y): mean ± SD	30.5 ± 4.9	31.2 ± 5.2	30.2 ± 4.7
Gestation age at invasive testing (wk): mean ± SD	27.5 ± 3.6	26.3 ± 3.1	27.9 ± 3.7
*Specimen*			
AF n (%)	81 (54.0%)	31 (79.5%)	50 (45.1%)
CB n (%)	69 (46.0%)	8 (20.5%)	61 (54.9%)

AF, amniotic fluid; CB, cord blood

### DNA extraction and CMA platforms

Genomic DNA was extracted from uncultured amniotic fluid, fetal cord blood using a QIAGEN kit (Qiagen, Hilden, Germany) according to the manufacturer's instructions. Single nucleotide polymorphism array (SNP array) was performed using Affymetrix CytoScan 750 K array (Affymetrix Inc., Santa Clara, CA, UA), which includes 200,000 probes for single nucleotide polymorphisms and 550,000 probes for copy number variations (CNVs) distributed across the entire human genome. Chromosome Analysis Suite software (Affymetrix) and human genome version GRCh37 (hg19) were used. A resolution was generally applied: gains or losses of ≥ 400 kb and regions of homozygosity (ROH) ≥ 10 Mb. Quality control was conducted by using the Median Absolute Pairwise Difference (MAPD) and SNP-QC score for copy number and SNP probes, respectively. Samples with MAPD > 0.25 and SNPQC < 15 for SNP array were excluded from the cohort. All CNVs were analyzed at the resolution of 100 kb of 50 markers and compared with in-house and national public CNV databases as follows: Database of Genomic Variants (DGV), Database of Chromosome Imbalance and Phenotype in Humans Using Ensemble Resources (DECIPHER), International Standards for Cytogenomic Arrays Consortium, and Online Mendelian Inheritance in Man (OMIM).

### CMA data interpretation

The CNVs were classified into five groups according to the American College of Medical Genetics (ACMG) definitions [12] and local database: pathogenic, benign, likely pathogenic, likely benign, and variants of uncertain significance (VOUS). Pathogenic/likely pathogenic CNVs were considered clinically significant findings. Parental CMA was recommended to determine the inheritance of CNVs.

### Routine karyotyping

Routine karyotyping consisted of cell culture and G-banded karyotyping was performed currently on cultured amniotic fluid and fetal cord blood according to the standard protocols in our laboratory. The karyotype was determined at a resolution of 320–500 bands level.

### Statistical analysis

The data were analyzed using SPSS software v26.0 (SPSS Inc., Chicago, IL, USA). Statistical comparisons were performed using the chi-square test, and *p* < 0.05 was considered statistically significant.

## Results

Among the 150 pregnancies with polyhydramnios, chromosomal aberrations were detected in 22 (14.7%) cases, including 3 cases of trisomy 21, 3 cases of trisomy 18, 9 cases of clinically significant CNVs, 3 cases of VOUS, 1 case of likely benign CNVs, and 3 cases of ROH with one of them being pathogenic. Therefore, the overall detection rate of clinically significant findings was 10.7% (16/150).

The distribution of clinically significant findings in isolated and non-isolated polyhydramnios pregnancies is summarized in Table 2, and the detailed results detected by CMA only are presented in Table 3.

**Table 2:** Distribution of clinically significant CMA findings in fetuses with isolated and non-isolated polyhydramnios

	Karyotype-detectable		CMA-detectable only		Total (%)
	T21 (%)	T18 (%)	CNVs (P + LP) (%)	LOH (%)	
Isolated (N = 39)	0, 0.0	0, 0.0	1, 2.6	0, 0.0	1, 2.6
Non-isolated(N = 111)	3, 2.7	3, 2.7	8, 7.2	1, 0.9	15, 13.5
soft markers (n = 59)	1, 1.7	0, 0.0	3, 5.1	0, 0.0	4, 6.8
Non-soft markers (n = 52)	2, 3.8	2, 3.8	5, 9.6	1, 1.9	10, 19.2
Total	3, 2.0	3, 2.0	9, 6.0	1, 0.7	16, 6.7

T21, trisomy 21; T18, trisomy 18; CNVs, copy number variants; P, pathogenic; LP, likely pathogenic; LOH, loss of heterozygosity

**Table 3:** Clinically significant CMA findings and ultrasound details in pregnancies with polyhydramnios and normal karyotype

Case Number	Gestational age (weeks)	Ultrasound findings	CMA results	Size	Inheritance	Pathogenicity category	Associated syndrome	Outcome
1	23+	Polyhydramnios	arr[GRCh37] 17p12(14,099,504–15,491,533) × 1	1.3 Mb	mat	Pathogenic	Hereditary Neuropathy With Liability to Pressure Palsies	Normal phenotype at 4-year-old follow-up
2	32+	Polyhydramnios, umbilical artery atresia	arr[GRCh37] 2q13(111,397,949–113,142,794) × 1	1.7 Mb	dn	Likely pathogenic	NV	Live birth with normal phenotype
3	34+	Polyhydramnios, ventricular septal defects, persistent left superior vena cava, nasal bone dysplasia	arr[GRCh37] 17p11.2(16,727,4900–20,433,723) × 1	3 Mb	dn	Pathogenic	Smith-Magenis Syndrome	TOP
4	19+	Polyhydramnios, pulmonary stenosis, strawberry like head, nuchal cystic lymphangioma	arr[GRCh37] 1p32.1p31.1(60,575,608–71,024,736) × 3	10.4 Mb	dn	Pathogenic	NV	TOP
5	26+	Polyhydramnios, bilateral ventriculomegaly, talipes	arr[GRCh37] 17p13.3p13.2(525–5,768,789) × 1	5.7 Mb	nd	Pathogenic	Miller-Dieker Syndrome	TOP
6	31+	Polyhydramnios, aberrant right subclavian artery	arr[GRCh37] 22q11.21(18,916,842–21,800,471) × 1	3.1 Mb	dn	Likely pathogenic	DiGeorge Syndrome	TOP
7	24+	Polyhydramnios, nasal bone dysplasia	arr[GRCh37] 16p12.2(21,816,542–22,710,614) × 1	994 Kb	dn	Likely pathogenic	NV	Live birth with normal phenotype
8	28+	Polyhydramnios, Hyperechogenic kidneys	arr[GRCh37] 17q12(34,822,465–36,404,555) × 1	1.58 Mb	dn	Likely pathogenic	17q12 Microdeletion Syndrome	Normal development at 3-year-old follow-up
9	25+	Twin pregnancy, polyhydramnios, right foot varusaberrant right subclavian artery	arr[GRCh37] 17p12p11.2(15,759,453–20,547,625) × 3	4.7 Mb	nd	Pathogenic	Potocki-Lupski Syndrome	ADHD, language disability at 3-year-old follow-up
10	32+	Polyhydramnios, FGR,VSD	arr[GRCh37] 15q14q21.3(35,077,111–54,347,324) hmz	19.2 Mb	mat upd	Pathogenic	Prader-Willi Syndrome	TOP

dn, de novo; nd, not detected; NV, not available; TOP, termination of pregnancy; FGR, fetal growth restriction; ADHD, attention deficit hyperactivity disorder; VSD, ventricular septal defect

In the isolated polyhydramnios group, one (2.6%) additional aberration by CMA was revealed. The only case showed a maternal 1.3 Mb deletion in the region of 17p12 (case 1, Table 2), which is related to neuropathy with liability to pressure palsies (HNPP) (#162500). The mother was prone to sprained ankles since the age of 35, and the child showed a normal phenotype before 4 years of age.

In the non-isolated group, the overall rate of clinically significant findings was 13.5% (15/111), which was not significantly higher than that in the isolated group (p > 0.05). The incremental yields of clinically significant results by CMA was 8.1% (9/111), including 8 cases of CNVs and 1 case of ROH. As shown in Table 3, 8 cases of CNVs (case 2–9) sized from 994 kb to 10.4 Mb, were detected in the non-isolated group, of which 5 were related to clinical syndromes: Smith-Magenis syndrome (#182290, case 3), Miller-Dieker syndrome (#247200, case 5), DiGeorge syndrome (#611867, case 6), 17q12 microdeletion syndrome (#614527, case 8), and Potocki–Lupski syndrome (#610883, case 9). In addition to CNVs, CMA yielded 1 case (case 10) of uniparental disomy (UPD). The case harbored a ROH of 19.2 Mb in region 15q14q21.3, which was finally confirmed to be composed of segmental UPiD and UPhD using the UPDtool. As a result, maternal UPD (15) related to Prader–Willi syndrome (PWS, **#**176270) was diagnosed, and the pregnancy was terminated. In this group, the incremental yields of clinically significant findings in pregnancies with soft markers and non-soft markers were 5.1% and 11.5%, respectively (*p* > 0.05).

Among the 19 fetuses with polyhydramnios from 17 twin pregnancies, 1 fetus demonstrated pathogenic CNVs (duplication on 17p12p11.2), which was related to Potocki–Lupski syndrome (case 9, Table 3). In addition to polyhydramnios, the fetus had aberrant right subclavian artery, and talipes; the other fetus of the twins had a normal ultrasound and normal CMA results. At the 3-year-old follow-up, the parents of the child with pathogenic CNVs mentioned that the child has attention deficit hyperactivity disorder (ADHD) and language disability.

## Discussion

In the past few years, CMA testing has been widely applied during prenatal development, especially for pregnancies with abnormal ultrasound findings [8, 13, 14]. In fetuses with ultrasound anomalies, CMA can detect an additional 5.2–10% of clinically significant aberrations compared to that by conventional karyotyping [14–16]. However, to the best of our knowledge, few studies have examined the association between polyhydramnios and genetic anomalies according to CMA analysis. Previous studies exploring the relationship between polyhydramnios and conventional karyotyping showed a 2.8% ± 3.7% pooled prevalence of chromosomal aberrations in pregnancies with idiopathic polyhydramnios, associated mainly with trisomy 21 and trisomy 18 [5, 6]. In our study, trisomy 21 and trisomy 18 were the only 2 chromosomal abnormalities detected by traditional karyotyping in non-isolated polyhydramnios pregnancies, and their overall incidence was 2.0%. These were detected in all fetuses of polyhydramnios, together with additional ultrasound abnormalities, especially structural abnormalities. Thus, aneuploidy was more likely to be detected when polyhydramnios was found in combination with additional ultrasound. A recent study with a large cohort of pregnancies with polyhydramnios undergoing CMA testing showed an additional 2.7% clinically significant abnormalities by CMA compared to that by conventional karyotyping [10]. In our present study, the incremental yield by CMA in isolated polyhydramnios group was 2.6%, similar to the 8.1% in the non-isolated group. With regard to the frequency of additional clinically significant findings within the non-isolated group, pregnancies with structural abnormalities were higher than pregnancies with soft markers, although not statistically significant. Therefore, CMA testing plays an important role in the etiological analysis of polyhydramnios, regardless of the presence of other ultrasound abnormalities.

Among the 10 additional clinically significant anomalies detected by CMA, 5 cases of CNVs were found on chromosome 17, including 17p12, 17p11.2, 17p13.3p13.2, and 17q12 deletions and 17p12p11.2 duplication, and they were related to HNPP, Smith–Magenis syndrome, Miller–Dieker syndrome, 17q12 microdeletion syndrome, and Potocki–Lupski syndrome, respectively. Among them, 17q12 microdeletion was more frequently reported in pregnancies with polyhydramnios [17–19]. Inefficient expression of *HNF1B* in the region of 17q12 is known to be a predominant factor leading to renal disease, which may result in fetal polyuria and polyhydramnios. The most common ultrasound finding in fetuses with 17q12 deletion was hyperechogenic kidneys [20], which was also present in our case. Thus, a view has been proposed when hyperechogenic kidney and polyhydramnios were observed prenatally, a possible diagnosis of 17q12 deletion should be considered [18, 19]. The 17q12 deletion is thought to be one of the most common microdeletions found in children with unexplained developmental delay [21] and may be associated with learning difficulties and autism [22, 23]. The child in our study manifested a normal phenotype at the 3-year-old follow-up, but long-term follow-up is still needed to accurately assess the prognosis. In addition to CNVs, a ROH of 15q14q21.3 detected in a polyhydramnios fetus with FGR was confirmed to be maternal UPD (15), which would result in PWS. Gross et al. [24] reported polyhydramnios in 43% of PWS pregnancies and a combination of polyhydramnios and FGR in 34% of PWS pregnancies. Geysenbergh et al. reported prenatal data of 11 children who were diagnosed with PWS and suggested that the combination of severe growth restriction and polyhydramnios can prompt clinicians to perform invasive tests for PWS diagnosis [25]. The only aberration detected in fetuses with isolated polyhydramnios was a maternal 1.3 Mb deletion on 17p12, which is a well-known pathogenic aberration related to HNPP. HNPP generally manifested as nerve disease after the second or third decade [26], and it has never been reported in previous reports of polyhydramnios. Therefore, the deletion was not a causative but an incidental finding.

Of note, most previous studies did not include twin pregnancies because of potentially confounding factors. In the present study, one or two fetuses with polyhydramnios from twin pregnancies without apparent twin-to-twin transfusion syndrome were included. In a pair of twins, one fetus with polyhydramnios developed an aberrant right subclavian artery and talipes and was revealed to have a duplication of 17p12p11.2 related to Potocki–Lupski syndrome, while the other fetus had normal ultrasound and CMA results. Potocki–Lupski syndrome is known to be associated with multiple congenital abnormalities, including developmental delays, autistic features, and certain structural anomalies, with cardiovascular being the most common [27, 28]. Both aberrant right subclavian artery and talipes have been previously reported in cases of PTLS [29–31], but polyhydramnios has not been reported before. The abnormal phenotype of ADHD and language disability after birth confirmed the diagnosis of Potocki–Lupski syndrome.

We acknowledged the following limitations of this study. First, the sample size was small, especially in the isolated group. Second, owing to missing data regarding the AFI/MVP, we could not perform an analysis on the risk of clinically significant CMA aberrations according to different degrees of polyhydramnios.

In conclusion, non-isolated polyhydramnios is associated with several genetic syndromes involving aneuploidy syndrome, microduplication syndrome, and microdeletion syndrome. CMA testing should be recommended in pregnancies with non-isolated polyhydramnios regardless of singleton or twin pregnancies. Our limited results suggest insufficient evidence to recommend CMA in pregnancies with isolated polyhydramnios.

## Supplementary Information

**Additional file 1**. Details of all 150 pregnancies with polyhydramnios.

## Data Availability

All data generated or analysed during this study are included in this published article and its Additional file 1.

## Abbreviations

CMA: Chromosomal microarray analysis
MVP: Maximum vertical pocket depth
AFI: Amniotic fluid index
CNVs: Copy number variations
DGV: Database of Genomic Variants
VOUS: Variants of uncertain significance
ROH: Loss-of heterozygosity
UPD: Uniparental disomy
TOP: Termination of pregnancy
FGR: Fetal growth restriction
ADHD: Attention deficit hyperactivity disorder
VSD: Ventricular septal defect

## References

[1] Dashe JS, McIntire DD, Ramus RM, Santos-Ramos R, Twickler DM (2002). Hydramnios: anomaly prevalence and sonographic detection. Obstet Gynecol.

[2] Magann EF, Chauhan SP, Doherty DA, Lutgendorf MA, Magann MI, Morrison JC (2007). A review of idiopathic hydramnios and pregnancy outcomes. Obstet Gynecol Surv.

[3] Schrimmer DB, Moore TR (2002). Sonographic evaluation of amniotic fluid volume. Clin Obstet Gynecol.

[4] Moise KJ (2013). Toward consistent terminology: assessment and reporting of amniotic fluid volume. Semin Perinatol.

[5] Lee JF, Wang KK, Lan CC (1996). Risk of fetal chromosomal abnormalities in idiopathic polyhydramnios. Zhonghua Yi Xue Za Zhi (Taipei).

[6] Sagi-Dain L, Sagi S (2015). Chromosomal aberrations in idiopathic polyhydramnios: a systematic review and meta-analysis. Eur J Med Genet.

[7] Hillman SC, McMullan DJ, Hall G, Togneri FS, James N, Maher EJ, Meller CH, Williams D, Wapner RJ, Maher ER, Kilby MD (2013). Use of prenatal chromosomal microarray: prospective cohort study and systematic review and meta-analysis. Ultrasound Obst Gynecol.

[8] Vanakker O, Vilain C, Janssens K, Van der Aa N, Smits G, Bandelier C, Blaumeiser B, Bulk S, Caberg JH, De Leener A, De Rademaeker M, de Ravel T, Desir J, Destree A, Dheedene A, Gaillez S, Grisart B, Hellin AC, Janssens S, Keymolen K, Menten B, Pichon B, Ravoet M, Revencu N, Rombout S, Staessens C, Van Den Bogaert A, Van Den Bogaert K, Vermeesch JR, Kooy F, Sznajer Y, Devriendt K (2014). Implementation of genomic arrays in prenatal diagnosis: the Belgian approach to meet the challenges. Eur J Med Genet.

[9] Bulletin P (2016). Practice bulletin no. 162: prenatal diagnostic testing for genetic disorders. Obstet Gynecol.

[10] Sagi-Dain L, Singer A, Falik-Zaccai T, Peleg A, Bar-Shira A, Feingold-Zadok M, Ben Shachar S, Maya I (2021). The effect of polyhydramnios degree on chromosomal microarray results: a retrospective cohort analysis of 742 singleton pregnancies. Arch Gynecol Obstet.

[11] Donnelly JC, Platt LD, Rebarber A, Zachary J, Grobman WA, Wapner RJ (2014). Association of copy number variants with specific ultrasonographically detected fetal anomalies. Obstet Gynecol.

[12] South ST, Lee C, Lamb AN, Higgins AW, Kearney HM (2013). ACMG standards and guidelines for constitutional cytogenomic microarray analysis, including postnatal and prenatal applications: revision 2013. Genet Med.

[13] Obstetricians ACo, Genetics GCo: Committee Opinion No. 581: the use of chromosomal microarray analysis in prenatal diagnosis. Obstet Gynecol. 2013;122(6):1374–1377. 10.1097/01.AOG.0000438962.16108.d1 24264715

[14] de Wit MC, Srebniak MI, Govaerts LC, Van Opstal D, Galjaard RJ, Go AT (2014). Additional value of prenatal genomic array testing in fetuses with isolated structural ultrasound abnormalities and a normal karyotype: a systematic review of the literature. Ultrasound in obstet Gynecol.

[15] Grati FR, Molina Gomes D, Ferreira JC, Dupont C, Alesi V, Gouas L, Horelli-Kuitunen N, Choy KW, Garcia-Herrero S, de la Vega AG, Piotrowski K, Genesio R, Queipo G, Malvestiti B, Herve B, Benzacken B, Novelli A, Vago P, Piippo K, Leung TY, Maggi F, Quibel T, Tabet AC, Simoni G, Vialard F (2015). Prevalence of recurrent pathogenic microdeletions and microduplications in over 9500 pregnancies. Prenat Diagn.

[16] Srebniak MI, Boter M, Oudesluijs GO, Cohen-Overbeek T, Govaerts LCP, Diderich KEM, Oegema R, Knapen MFCM, van de Laar IMBH, Joosten M, Van Opstal D (2012). Galjaard R-JH: genomic SNP array as a gold standard for prenatal diagnosis of foetal ultrasound abnormalities. Mol Cytogenet.

[17] Sagi-Dain L, Maya I, Reches A, Frumkin A, Grinshpun-Cohen J, Segel R, Manor E, Khayat M, Tenne T, Banne E, Shalata A, Yonath H, Berger R, Singer A, Ben-Shachar S (2018). Chromosomal microarray analysis results from pregnancies with various ultrasonographic anomalies. Obstet Gynecol.

[18] Jones GE, Mousa HA, Rowley H, Houtman P, Vasudevan PC (2015). Should we offer prenatal testing for 17q12 microdeletion syndrome to all cases with prenatally diagnosed echogenic kidneys? Prenatal findings in two families with 17q12 microdeletion syndrome and review of the literature. Prenat Diagn.

[19] Zhang Y, Liu Y, Yan L, Zhuang D, Li H (2021). Prenatal diagnosis and genetic analysis of two fetuses with paternally derived 17q12 microdeletions. Zhonghua Yi Xue Yi Chuan Xue Za Zhi.

[20] Gondra L, Décramer S, Chalouhi GE, Muller F, Salomon R, Heidet L (2016). Hyperechogenic kidneys and polyhydramnios associated with HNF1B gene mutation. Pediatric Nephrol (Berlin, Germany).

[21] Moreno-De-Luca D, Mulle JG, Kaminsky EB, Sanders SJ, Myers SM, Adam MP, Pakula AT, Eisenhauer NJ, Uhas K, Weik L, Guy L, Care ME, Morel CF, Boni C, Salbert BA, Chandrareddy A, Demmer LA, Chow EWC, Surti U, Aradhya S, Pickering DL, Golden DM, Sanger WG, Aston E, Brothman AR, Gliem TJ, Thorland EC, Ackley T, Iyer R, Huang S, Barber JC, Crolla JA, Warren ST, Martin CL, Ledbetter DH (2011). Deletion 17q12 Is a recurrent copy number variant that confers high risk of autism and schizophrenia. Am J Hum Genet.

[22] Bernardini L, Gimelli S, Gervasini C, Carella M, Baban A, Frontino G, Barbano G, Divizia MT, Fedele L, Novelli A, Béna F, Lalatta F, Miozzo M, Dallapiccola B (2009). Recurrent microdeletion at 17q12 as a cause of Mayer–Rokitansky–Kuster–Hauser (MRKH) syndrome: two case reports. Orphanet J Rare Dis.

[23] Chabane N (2010). Autism in three patients with cystic or hyperechogenic kidneys and chromosome 17q12 deletion. Nephrol Dial Transplant.

[24] Gross N, Rabinowitz R, Gross-Tsur V, Hirsch HJ, Eldar-Geva T (2015). Prader-Willi syndrome can be diagnosed prenatally. Am J Med Genet Part A.

[25] Geysenbergh B, De Catte L, Vogels A (2011). Can fetal ultrasound result in prenatal diagnosis of Prader-Willi syndrome?. Genet Counsel (Geneva, Switzerland).

[26] Meretoja P, Silander K, Kalimo H, Aula P, Meretoja A, Savontaus ML (1997). Epidemiology of hereditary neuropathy with liability to pressure palsies (HNPP) in south western Finland. Neuromuscul Disord.

[27] Potocki L, Bi W, Treadwell-Deering D, Carvalho CM, Eifert A, Friedman EM, Glaze D, Krull K, Lee JA, Lewis RA, Mendoza-Londono R, Robbins-Furman P, Shaw C, Shi X, Weissenberger G, Withers M, Yatsenko SA, Zackai EH, Stankiewicz P, Lupski JR (2007). Characterization of Potocki-Lupski syndrome (dup(17)(p11.2p11.2)) and delineation of a dosage-sensitive critical interval that can convey an autism phenotype. Am J Hum Genet.

[28] Yusupov R, Roberts AE, Lacro RV, Sandstrom M, Ligon AH (2011). Potocki-Lupski syndrome: an inherited dup(17)(p11.2p11.2) with hypoplastic left heart. Am J Med Gen Part A.

[29] Bravo C, Gamez F, Perez R, Aguaron A, De Leon-Luis J (2013). Prenatal diagnosis of Potocki-Lupski syndrome in a fetus with hypoplastic left heart and aberrant right subclavian artery. J Perinatol.

[30] Lehman WB, Dhanaraj D, Moran E, Pappas JG (2015). Potocki-Lupski syndrome in conjunction with bilateral clubfoot. J Pediatric Orthop Part B.

[31] Dhanaraj D, Chu A, Pappas JG, Moran E, Lehman WB (2015). Potocki-Lupski syndrome in conjunction with bilateral clubfoot. J Pediatr Orthop B.

