# Supplementary material for: Evaluation of genetic variants using chromosomal microarray analysis for fetuses with polyhydramnios
Source: BMC Med Genomics. 2022 Mar 30;15:73. doi: 10.1186/s12920-022-01224-w (PMC8966299; doi:10.1186/s12920-022-01224-w)
Supplement: Supplementary file 1 — Additional file 1. Details of all 150 pregnancies with polyhydramnios. [file 12920_2022_1224_MOESM1_ESM.docx]

| **Cases** | **maternal age** | **gestational age** | **Ultrasound findings** | **Karyotypes** | **CMA results** |
| --- | --- | --- | --- | --- | --- |
| E1382 | 37 | 30 | Polyhydramnios, increased nuchal fold | 46,X,inv(Y)pat | N |
| E1880 | 27 | 26 | Polyhydramnios, bilateral ventriculomegaly, talipes | 46,XY | arr[GRCh37] 17p13.3p13.2(525-5,768,789)×1 |
| E1936 | 26 | 25 | Polyhydramnios, increased nuchal fold | 46,XY | N |
| E1943 | 26 | 32 | Polyhydramnios, aortic stenosis, increased cardiothoracic ratio | 46,XY | N |
| E2005 | 40 | 32 | Polyhydramnios, FGR | 46,XX | arr[GRCh37] 15q14q21.3(35,077,111-54,347,324) hmz |
| E2010 | 22 | 32 | Polyhydramnios, Single umbilical artery | 46,XX | N |
| E2034 | 27 | 26 | Polyhydramnios | 46,XX,inv(12)(p11.2q21.2)dn | N |
| E2078 | 39 | 28 | Polyhydramnios | 46,XY | N |
| E2085 | 37 | 34 | Polyhydramnios, short femur | 46,XY | N |
| E2143 | 21 | 32 | Polyhydramnios, FGR | 46,X,inv(Y)(p11.2q11.2) pat | N |
| E2153 | 27 | 32 | Polyhydramnios, increases nuchal translucency | 46,XX | N |
| E2157 | 28 | 31 | Polyhydramnios, Ventriculomegaly, renal pelvis separation, Echogenic bowel | 46,XY | N |
| E2167 | 32 | 26 | Polyhydramnios | 46,XY | N |
| E2198 | 26 | 32 | Polyhydramnios, FGR | 46,XY | N |
| E2212 | 34 | 32 | Polyhydramnios, short FL, short HL, Dextrocardia, Pleural Effusion | 46,XY | N |
| E2225 | 33 | 27 | Polyhydramnios, ventricular septal defects | 46,XX | N |
| E2228 | 30 | 29 | Polyhydramnios, spinal dysplasia, right Overlapping Fingers, rocker-bottom foot | 46,XX | N |
| E2297 | 37 | 25 | Polyhydramnios, Echogenic bowel | 46,XY | N |
| E2305 | 37 | 32 | Polyhydramnios, tricuspid regurgitation, lower FL/BPD, lower FL/HC, tricuspid regurgitation | 46,XY | N |
| E2312 | 27 | 28 | Polyhydramnios, increased NF, paranasal thickness thickening | 46,XY | N |
| E2375 | 38 | 33 | Polyhydramnios, lower gastrointestinal atresia | 46,XY | N |
| E2413 | 37 | 31 | polyhydramnios | 46,XX | N |
| E2431 | 28 | 35 | Polyhydramnios, Ventriculomegaly, suspicious intracranial Hemorrhages | 46,XY | N |
| E2432 | 28 | 30 | polyhydramnios, renal collecting system separation | 46,XY | N |
| E2498 | 28 | 27 | Polyhydramnios, mitral regurgitation, Abnormal blood flow in the middle cerebral artery, tricuspid regurgitation | 46,XX | N |
| E2505 | 37 | 30 | Polyhydramnios, tricuspid regurgitation | 46,XY | N |
| E2513 | 32 | 33 | Polyhydramnios, Pleural Effusion | 46,XY | N |
| E2518 | 35 | 33 | Polyhydramnios, abdominal effusion, Gastrointestinal Tract obstruction | 46,XX | N |
| E2561 | 30 | 29 | Polyhydramnios, shorter FL, Abnormal umbilical artery blood flow | 46,XY | N |
| E2590 | 31 | 27 | Polyhydramnios, tricuspid regurgitation | 46,XY | N |
| E2600 | 27 | 27 | Polyhydramnios, shorter HL, shorter FL | 46,XY | N |
| E2647 | 28 | 32 | duodenal atresia/stenosis, Polyhydramnios | 46,XX | N |
| E2690 | 34 | 31 | Polyhydramnios, VENTRICULAR SEPTAL DEFECTS , Paranasal thickness thickening, NF thickening | 46,XX | N |
| E2707 | 27 | 32 | Cardiomegaly, polyhydramnios | 46,XY | N |
| E2725 | 36 | 27 | right Ventriculomegaly, polyhydramnios | 46,XY | N |
| E2732 | 26 | 33 | Echogenic bowel, Polyhydramnios | 46,XY | N |
| E2752 | 29 | 31 | nuchal cystic lymphangioma, Polyhydramnios, renal pelvis separation | 46,XY | N |
| E2759 | 30 | 29 | Ventricular septal defects , Polyhydramnios; tricuspid regurgitation | 46,XY | N |
| E2794 | 30 | 32 | posterior fossa pool broadening, polyhydramnios, renal pelvis separation | 46,XY | N |
| E2828 | 29 | 27 | left echogenic intra-cardiac focus, left Ventriculomegaly, Polyhydramnios | 46,XY | N |
| E2864 | 32 | 32 | left ventriculomegaly, Echogenic bowel, Polyhydramnios | 46,XY | N |
| E2884 | 28 | 33 | Left Ventriculomegaly, Polyhydramnios | 46,XY | N |
| E2910 | 28 | 30 | Polyhydramnios, posterior fossa pool broadening | 46,XY | N |
| E2925 | 27 | 28 | polyhydramnios, Persistent right umbilical vein | 46,XY | N |
| E2982 | 31 | 32 | Polyhydramnios | 46,XY | N |
| E3012 | 23 | 26 | renal pelvis separation, left echogenic intra-cardiac focus, polyhydramnios | 46,XX | N |
| E3021 | 26 | 27 | duodenal atresia/stenosis, Polyhydramnios | 46,XX | N |
| E3050 | 24 | 30 | Echogenic bowel, Polyhydramnios | 46,XY | N |
| E3092 | 33 | 27 | Echogenic bowel, polyhydramnios | 46,XY | N |
| E3107 | 25 | 30 | shorter HL, FL, polyhydramnios | 46,XY | N |
| E3184 | 35 | 28 | Polyhydramnios, Hyper-echogenic kidneys | 46,XX | arr[GRCh37] 17q12(34,822,465-36,404,555)×1 dn |
| E3205 | 33 | 29 | 左echogenic intra-cardiac focus, tricuspid regurgitation, Polyhydramnios | 46,XY | N |
| E3248 | 29 | 32 | FGR？Polyhydramnios | 46,XX | N |
| E3292 | 37 | 29 | Echogenic bowel, polyhydramnios | 46,XX | N |
| E3402 | 22 | 32 | Polyhydramnios | 46,XY | N |
| E3522 | 32 | 29 | Polyhydramnios | 46,XX | N |
| E3528 | 26 | 27 | echogenic intra-cardiac focus, Polyhydramnios | 46,XX | N |
| E3534 | 29 | 30 | Ventricular septal defects , esophageal atresia, Polyhydramnios | 46,XX | N |
| E3558 | 36 | 30 | Polyhydramnios, bilateral Ventriculomegaly | 46,XY | N |
| E3565 | 33 | 31 | Increased cardiothoracic ratio, polyhydramnios | 46,XY | N |
| E3594 | 34 | 31 | Polyhydramnios, esophageal atresia | 46,XY | N |
| E3609 | 32 | 28 | echogenic intra-cardiac focus, tricuspid regurgitation, polyhydramnios | 46,XY | N |
| E3640 | 34 | 34 | Polyhydramnios, umbilical artery atresia | 46,XX | arr[GRCh37] 17p11.2(16,727,4900-20,433,723)×1 dn |
| E3642 | 34 | 30 | Echogenic bowel, polyhydramnios, shorter HL, FL | 46,XY | N |
| E3695 | 18 | 33 | aberrant right subclavian artery; polyhydramnios | 46,XX | N |
| E595 | 22 | 30 | shorter FL、shorter HL; Polyhydramnios | 47,XY,+21 | arr[GRCh37] 21×3 |
| E661 | 32 | 30 | duodenal atresia/stenosis, Polyhydramnios, bilateral hyperechoic kidneys, Polyhydramnios | 47,XY,+21 | arr[GRCh37] 21×3 |
| E687 | 20 | 29 | Double Outlet Right Ventricle, ventricular septal defects , Diaphragmatic hernia, overlapping fingers, Single umbilical artery, Polyhydramnios | 47,XY,+18 | arr[GRCh37] 18×3 |
| E907 | 35 | 31 | Single Atrium, single ventricle heart. aortic stenosis, persistent left superior vena cava, posterior fossa pool broadening, strawberry-like head, overlapping fingers, Single umbilical artery, Polyhydramnios | 47,XX,+18 | arr[GRCh37] 18×3 |
| G7510 | 43 | 24 | smaller AC,FL,HL, ventricular septal defects , left Ventriculomegaly, choroid plexus cyst enlarged gallbladder; Polyhydramnios | 47,XX,+18 | arr[GRCh37] 18×3 |
| G8400 | 26 | 24 | Bilateral Ventriculomegaly, enlarged gallbladder; Polyhydramnios | 46,XY | N |
| G9237 | 26 | 31 | persistent left superior vena cava, polyhydramnios | 46,XY | N |
| P1133 | 28 | 23 | left echogenic intra-cardiac focus, Echogenic bowel, Polyhydramnios | 46,XY | N |
| P1268 | 23 | 26 | Polyhydramnios; Twin pregnancy | 46,XX | arr[GRCh37] 14q12(25,364,014-26,860,808)×3 |
| P2015 | 26 | 23 | Isolated polyhydramnios | 46,XX | arr[GRCh37] 17p12(14,099,504-15,491,533)×1 mat |
| P2104 | 31 | 26 | Twin pregnancy, polyhydramnios, right foot Varus aberrant right subclavian artery | 46,XY | arr[GRCh37] 17p12p11.2(15,759,453-20,547,625)×3 dn |
| P2223 | 32 | 23 | Twin pregnancy, Polyhydramnios | 46,XX | N |
| P2452 | 37 | 23 | polyhydramnios, persistent left superior vena cava | 46,XY | N |
| P2462 | 28 | 26 | duodenal atresia/stenosis, tricuspid regurgitation, small amount of Pericardial Effusion, abdominal effusion, and pleural Effusion; Polyhydramnios | 46,XY | N |
| P2644 | 36 | 26 | Polyhydramnios | 46,XY | N |
| P2706 | 29 | 23 | Polyhydramnios, choroid plexus cyst | 46,XX | N |
| P3015 | 35 | 24 | pulmonary stenosis, Left ventricular wall thickening, strawberry-like head, nuchal cystic lymphangioma, Polyhydramnios; Twin pregnancy | 46,XX | N |
| P3021 | 39 | 19 | Polyhydramnios, pulmonary stenosis, strawberry like head, nuchal cystic lymphangioma | 46,XY | arr[GRCh37]1p32.1p31.1(60,575,608-71,024,736)×3 dn |
| P3197 | 41 | 25 | Polyhydramnios | 46,XY | N |
| P3290 | 26 | 23 | Brain atrophy, Echogenic bowel, Polyhydramnios; Twin pregnancy | 46,XY | arr[GRCh37] 9p21.1(28,563,687-30,820,392)×1 |
| P3349 | 29 | 24 | Polyhydramnios | 46,XY | N |
| P3497 | 36 | 20 | Polyhydramnios | 46,XY | N |
| P3787 | 31 | 20 | Bilateral Ventriculomegaly, Arachnoid Cysts , Agenesis of Corpus Callosum, Cerebellar vermis dysplasia, Polyhydramnios | 46,XY | N |
| P4326 | 34 | 31 | Polyhydramnios, lower AC, lower FL, Single umbilical artery, | 46,XY | N |
| P4758 | 27 | 25 | Polyhydramnios, shorter FL, shorter HL, | 46,XX | N |
| P488 | 40 | 24 | Polyhydramnios | 46,XY | N |
| P4926 | 26 | 25 | Polyhydramnios | 46,XY | N |
| P4999 | 29 | 25 | Pleural Effusion,abdominal effusion, shorter FL, bilateral hyperechoic kidneys , tricuspid regurgitation, polyhydramnios | 46,XX | N |
| P5128 | 35 | 23 | polyhydramnios, esophageal atresia | 46,XY | N |
| P6012 | 25 | 23 | polyhydramnios, right Ventriculomegaly | 46,X,inv(Y)(p11.2q11.2) pay | N |
| P6062 | 32 | 23 | Polyhydramnios; Single umbilical artery, right foot varus, Twin pregnancy;SIUGRF1 | 46,XX | N |
| P6147 | 30 | 27 | Polyhydramnios, Twin pregnancy | 46,XX | N |
| P6718 | 30 | 24 | Polyhydramnios, intralobar pulmonary | 46,XX | N |
| P7188 | 36 | 23 | Polyhydramnios, Twin pregnancy; | 46,XY | N |
| P7189 | 36 | 23 | Polyhydramnios, Twin pregnancy; | 46,XX | N |
| P7447 | 32 | 24 | polyhydramnios; tricuspid regurgitation, | 46,XY | N |
| P7522 | 30 | 23 | Polyhydramnios, Lower FL/BPD, lower FL/HC; ventricular septal defects , overriding aorta, pulmonary artery stenosis, Nasal bone dysplasia, duodenal atresia/stenosis | 47,XY,+21 | arr[GRCh37] 21×3 |
| P7817 | 32 | 24 | Polyhydramnios, nasal bone dysplasia | 46,XX | arr[GRCh37] 16p12.2(21,816,542-22710,614)×1 pat |
| P7870 | 33 | 24 | Polyhydramnios, pulmonary stenosis, tricuspid regurgitation | 46,XY | N |
| P8103 | 35 | 25 | Polyhydramnios, | 46,XY | N |
| P8109 | 34 | 25 | Polyhydramnios, Persistent right umbilical vein | 46,XY | arr[GRCh37] 7p21.2(13,855,150-16,262,103)x1 de novo |
| P8333 | 39 | 28 | Ventricular septal defects , Polyhydramnios | 46,XY | N |
| P8815 | 28 | 24 | Polyhydramnios; Twin pregnancy, ventricular septal defects , echogenic intra-cardiac focus, | 46,XY | N |
| P8976 | 27 | 26 | Polyhydramnios; Twin pregnancy | 46,XX | N |
| P8977 | 27 | 26 | Polyhydramnios; Twin pregnancy | 46,XX | N |
| P9002 | 25 | 25 | Polyhydramnios | 46,XX | N |
| P9107 | 27 | 24 | Polyhydramnios | 46,XY,21pss | N |
| P9145 | 24 | 26 | polyhydramnios | 46,XX | N |
| P9339 | 29 | 25 | Polyhydramnios, tricuspid regurgitation, | 46,XY | N |
| P9362 | 32 | 22 | polyhydramnios, Gastric duplication | 46,XX | N |
| P9461 | 31 | 19 | Polyhydramnios, choroid plexus cysts | 46,XY | N |
| P9472 | 30 | 32 | Polyhydramnios, umbilical artery atresia | 46,XY | arr[GRCh37] 2q13(111,397,949-113,142,794)×1 dn |
| P9578 | 30 | 28 | Polyhydramnios | 46,XY | N |
| P9718 | 30 | 32 | Polyhydramnios, Hydrops Fetalis, enlarged liver | 46,XX | N |
| R1050 | 37 | 32 | Polyhydramnios, upper Gastrointestinal tract obstruction | 46,XX | N |
| R1196 | 17 | 29 | Polyhydramnios; echogenic intra-cardiac focus, Nasal bone dysplasia | 46,XY | N |
| R1228 | 31 | 22 | Polyhydramnios, echogenic intra-cardiac focus, ventricular septal defects | 46,XX | N |
| R1289 | 27 | 26 | Polyhydramnios, echogenic intra-cardiac focus, | 46,XY | N |
| R1392 | 34 | 20 | Polyhydramnios | 46,XY | N |
| R1727 | 33 | 23 | Polyhydramnios, twin pregnancy | 46,XY | N |
| R2131 | 24 | 25 | Polyhydramnios, echogenic intra-cardiac focus, tricuspid regurgitation, twin pregnancy | 46,XY | N |
| R2245 | 30 | 29 | Polyhydramnios | 46,XX | N |
| R2451 | 34 | 30 | polyhydramnios, Nasal bone dysplasia | 46,XY | N |
| R2555 | 33 | 26 | Polyhydramnios, echogenic intra-cardiac focus, | 46,XY | N |
| R2638 | 31 | 28 | Polyhydramnios, twin pregnancy | 46,XY | arr[GRCh37]1p36.21(15,728,288-31781,279)×2 hmz, 4p15.2p11(25,981,952-49,063,479)×2 hmz |
| R2639 | 33 | 23 | Polyhydramnios; renal pelvis separation ,ventricular septal defects | 46,XY | N |
| R268 | 26 | 28 | Polyhydramnios; echogenic intra-cardiac focus | 46,XY | arr[GRCh37] 20p13(267,311-961,751)×3 mat |
| R2754 | 41 | 28 | Polyhydramnios | 46,XY | arr[GRCh37]2q31.1q32.2(177,156,393-190,026,211)×2 hmz |
| R2985 | 28 | 23 | Polyhydramnios, echogenic intra-cardiac focus, tricuspid regurgitation | 46,XX | N |
| R3072 | 27 | 25 | Polyhydramnios, upper Gastrointestinal tract obstruction | 46,XX | N |
| R3082 | 25 | 30 | Polyhydramnios | 46,XY | N |
| R3116 | 30 | 30 | polyhydramnios, Echogenic bowel | 46,XY | N |
| R3218 | 30 | 23 | Polyhydramnios, twin pregnancy, SIUGR | 46,XX | N |
| R560 | 33 | 24 | Polyhydramnios, twin pregnancy | 46,XY | N |
| R615 | 32 | 24 | polyhydramnios, Echogenic bowel | 46,XY | N |
| R767 | 26 | 31 | Polyhydramnios, aberrant right subclavian artery | 46,XY | arr[GRCh37] 22q11.21(18,916,842-21,800,471)×1 dn |
| R909 | 21 | 22 | Polyhydramnios | 46,XY | N |
| S111 | 28 | 30 | Polyhydramnios | 46,XX | N |
| S116 | 35 | 29 | Polyhydramnios | 46,XX | N |
| S119 | 35 | 31 | Polyhydramnios | 46,XY | N |
| S13 | 28 | 26 | Polyhydramnios, hydronephrosis | 46,XY | N |
| S56 | 28 | 26 | Polyhydramnios, increased nuchal thickness | 46,XX | N |
| S57 | 29 | 28 | Polyhydramnios | 46,XY | N |
| S60 | 30 | 27 | Polyhydramnios, esophageal atresia, Single umbilical artery | 46,XX | N |
| S79 | 35 | 30 | Polyhydramnios | 46,XX | N |

Abbreviation; N, normal; FGR, fetal growth restriction; HL, humerus length; FL, femur length; AC, Abdominal Circumference; SIUGR, selective intrauterine growth restriction,
